# Bioelectroventing: an electrochemical‐assisted bioremediation strategy for cleaning‐up atrazine‐polluted soils

**DOI:** 10.1111/1751-7915.12687

**Published:** 2017-06-23

**Authors:** Ainara Domínguez‐Garay, Jose Rodrigo Quejigo, Ulrike Dörfler, Reiner Schroll, Abraham Esteve‐Núñez

**Affiliations:** ^1^ University of Alcalá Alcalá de Henares Madrid Spain; ^2^ Helmholtz Zentrum München Múnich Germany; ^3^ IMDEA‐WATER Parque Tecnológico de la Universidad de Alcalá Madrid Spain

## Abstract

The absence of suitable terminal electron acceptors (TEA) in soil might limit the oxidative metabolism of environmental microbial populations. *Bioelectroventing* is a bioelectrochemical strategy that aims to enhance the biodegradation of a pollutant in the environment by overcoming the electron acceptor limitation and maximizing metabolic oxidation. Microbial electroremediating cells (MERCs) are devices that can perform such a *bioelectroventing*. We also report an overall profile of the ^14^C‐ATR metabolites and ^14^C mass balance in response to the different treatments. The objective of this work was to use MERC principles, under different configurations, to stimulate soil bacteria to achieve the complete biodegradation of the herbicide ^14^C‐atrazine (ATR) to ^14^ CO_2_ in soils. Our study concludes that using electrodes at a positive potential [+600 mV (versus Ag/AgCl)] ATR mineralization was enhanced by 20‐fold when compared to natural attenuation in electrode‐free controls. Furthermore, ecotoxicological analysis of the soil after the bioelectroventing treatment revealed an effective clean‐up in < 20 days. The impact of electrodes on soil bioremediation suggests a promising future for this emerging environmental technology.

## Introduction

Biodegradation is a major process in the complete mineralization of aromatic compounds in the environment and is considered an *in situ* treatment that avoids the costs derived from excavation and emission control, and therefore is considered a cheap and clean technology (Khan *et al*., 2004). However, only a certain proportion of these compounds can be degraded by the soil microbial community (Katayama *et al*., 2010), being considered as a process that is highly dependent on the presence of indigenous degrading microbes as well as several abiotic factors (soil properties, temperature, plant presence, soil moisture) which have considerable effects on the herbicide's sorption and bioavailability (Ngigi *et al*., 2011). In flooded soils under strong reductive conditions, the deficiency of suitable terminal electron acceptors (TEA) limits microbial respiration, and as a consequence, a variety of organic pollutants persist in waterlogged soils or sediments (Liu and Suflita, 1993). Traditional bioremediation techniques have overcome this TEA constrain supplying additional electron acceptors like oxygen (bioventing) (Kabelitz *et al*., 2009; Frutos *et al*., 2010), humic acids (Lovley, 2000) or nitrates (Yu *et al*., 2014), to stimulate microbial metabolism, but in addition to the costs of chemicals, concerns are raised due to potential secondary pollution, i.e., by nitrite (Pandey, 2012).

Terminal electron acceptors limitations can be alternatively overcome using electrically conductive material like the electrodes used in microbial electroremediating cell (MERC) (Rodrigo *et al*., 2014). These devices allow microorganisms to transfer electrons from oxidative metabolism to a soil‐buried electrode (anode). The anode is connected, through an external resistor, to a cathode placed in an aerobic environment where electrons are finally consumed by an electron acceptor such as oxygen (Tender *et al*., 2002; De Schamphelaire *et al*., 2008; Venkata Mohan *et al*., 2009; Wang *et al*., 2012a,b; Domínguez‐Garay *et al*., 2013). MERCs are bioelectrochemical variants of sediment microbial fuel cells (SMFCs), although they differ in their operational mode. While SMFCs aim to maximize power generation (Watts), MERCs aim to reach maximum current production (Amperes) by maximizing metabolic oxidation of organic/inorganic soil compounds and consequently the degradation rate by microorganisms (Rodrigo *et al*., 2014).

We have explored electrochemical‐assisted scenarios where oxidative metabolism can be enhanced to remove pollutants. Zhang *et al*. (2010) reported for first time that graphite electrodes could serve as an electron acceptor for the degradation of toluene and benzene in polluted slurries (Zhang *et al*., 2010). Since then, several studies have used microbial electrochemical system to enhance the biodegradation of pollutants of different chemical natures: petroleum hydrocarbons (Morris and Jin, 2012; Zhang *et al*., 2014; Daghio *et al*., 2016), PAHs (Chandrasekhar and V.M., 2012; Wang *et al*., 2012a,b; Yan *et al*., 2012; Rodrigo *et al*., 2014; Li and Yu, 2015; Li *et al*., 2015; Sherafatmand and Ng, 2015), phenol (Huang *et al*., 2011), nitrobenzene (Liang *et al*., 2014), pesticides (Yeh and Chen, 2006; Cao *et al*., 2015; Rodrigo Quejigo *et al*., 2016) and chlorinated organics (Yeh and Chen, 2006; Chun *et al*., 2013; Liu *et al*., 2013; Yu *et al*., 2016). Moreover, not just pollutant removal but an efficient clean‐up was demonstrated by ecotoxicological analysis of a DBT‐polluted soil after MERC treatment (Rodrigo *et al*., 2014). The same results were obtained by genotoxicological and phytotoxicological assays in atrazine‐polluted soils after stimulating the microbial oxidation with an electrode (Domínguez‐Garay *et al*., 2016).

The redox gradient in MERC is established spontaneously across the soil–water interphase as a result of spatially segmented reduction–oxidation reactions establishing an electron transport route between electrodes (Li and Yu, 2015). This electrode potential is typically negative and can be insufficient for effective transformation of recalcitrant compounds due to the high ohmic internal resistance of the system (Domínguez‐Garay *et al*., 2013). To overcome this limitation, several studies have applied an external voltage between the anode and cathode providing more favourable redox potentials for soil microorganisms (Aulenta *et al*., 2007; Schrotta *et al*., 2011; Chun *et al*., 2013). More recently, Rodrigo Quejigo *et al*. (2016) reported how fine‐tuning the electrode potential can have a strong impact on the pollutant mineralization (Rodrigo Quejigo *et al*., 2016).

Another pollutant that has been subject of study through bioelectrochemical strategies by MERCs is atrazine (2‐chloro‐4‐ethylamino‐6‐isopropyl amino‐1,3,5‐triazine) (Domínguez‐Garay *et al*., 2016). Atrazine degradation is mainly a result of microbial activity (Mudhoo and Garg, 2011). A large variety of microbes such as *Pseudomonas* sp ADP (de Souza *et al*., 1998)*, Agrobacterium radiobacter* J14a (Struthers *et al*., 1998) or *Nocardioides* (Topp *et al*., 2000) degrade atrazine through co‐metabolic processes that lead to the formation and accumulation of atrazine‐derived metabolites. Although atrazine was banned in the European Union in 2003 (Bethsass and Colangelo, 2006), it is still widely used around the world due to its low cost and high effectiveness for control of weeds in crops such as corn and sorghum. Numerous studies have indicated that atrazine inhibits growth and photosynthesis of freshwater algae and algal responses to atrazine vary widely depending upon concentrations, time of exposure and algal species tested (Tang *et al*., 1997; Weiner *et al*., 2004).

The main objective of our work was to stimulate soil native bacteria by *bioelectroventing,* in order to enhance the complete biodegradation of ^14^C‐atrazine to ^14^CO_2_. Our ecotoxicological analysis has proven that electrode potentials as high as 600 mV (versus Ag/AgCl) accelerate the cleaning‐up of ATR‐polluted soils.

## Results and discussion

### Bioelectrochemical interrogation of the microbial redox activity

**Figure 1:**
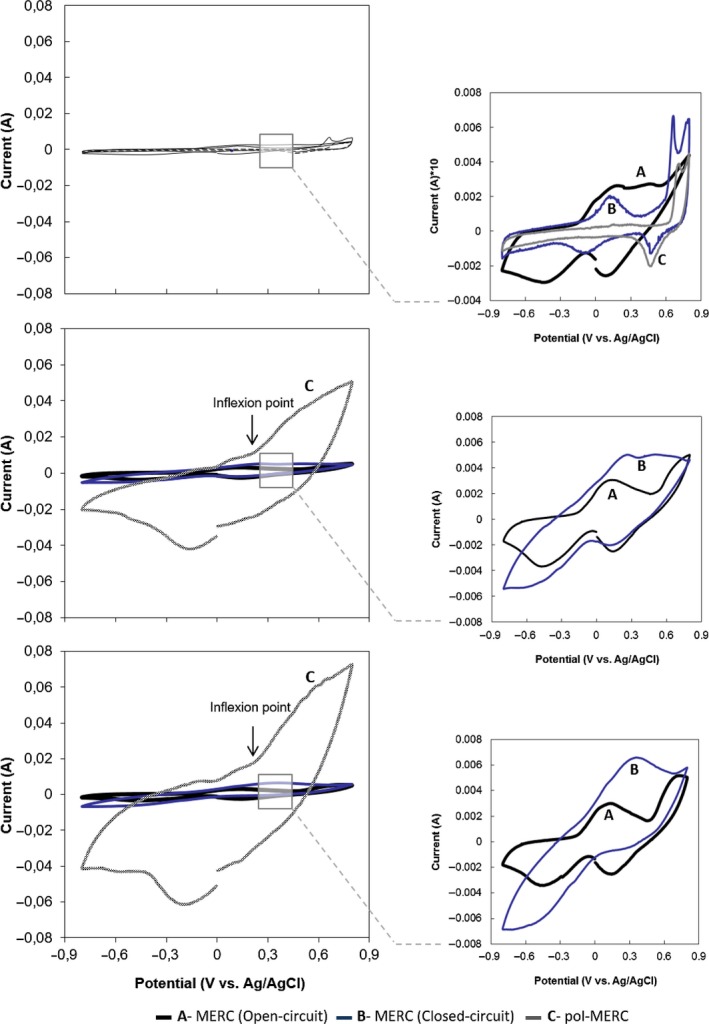
Cyclic voltammetry analysis at different incubation periods (1, 7 and 20 days) for different configurations A. MERC (open‐circuit), B. MERC (closed‐circuit) and C. pol‐MERC. CVs were recorded at 1 mV s^−1^ from 0.8 and −0.8V and back to 0.8V (versus Ag/AgCl). The graphics at the right column focus closely on the voltammetries.

**Figure 2:**
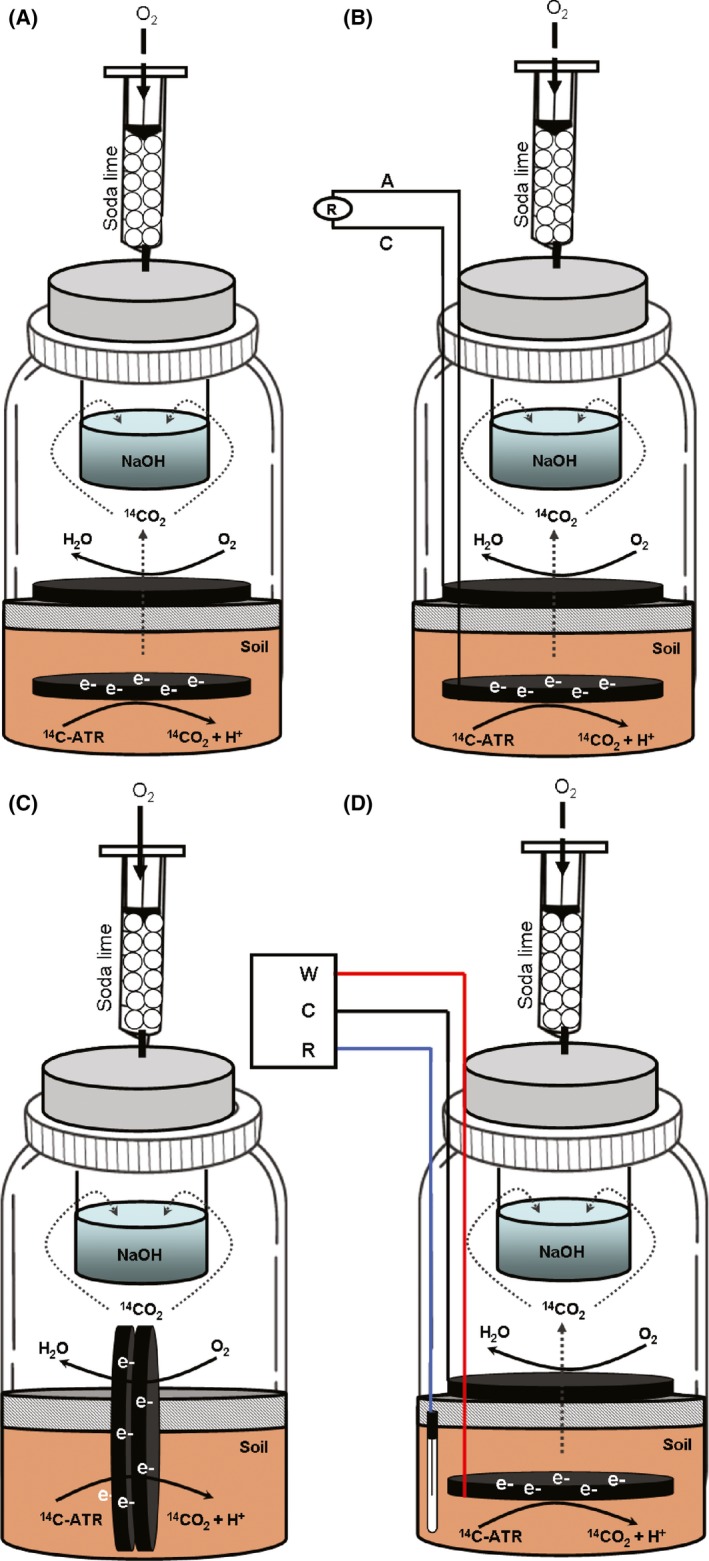
Experimental design for monitoring the ^14^C‐ATR mineralization under different configurations. A. MERC under open‐circuit conditions (anode and cathode disconnected), B. MERC under closed‐circuit conditions (anode and cathode connected by a 5 Ω external resistor), C. snorkel configuration (carbon felt electrodes vertically oriented, partly buried in the soil and partly in contact with the flooded water body), D. pol‐MERC, a three‐electrode system controlled by a potentiostat for polarizing the anode (working electrode) at 0.6 V versus Ag/AgCl reference electrode.

In order to evaluate the degradation activity by soil microorganisms exposed to different experimental conditions, to understand the relationship between the cumulative mineralization and the increased biological activity, several cyclic voltammetries (CVs) were taken along the experimental phase to study the electron transfer interactions between soil microorganisms and anodes (Fig. 1). The CVs after 24 h revealed an inflexion point at approximately −400 and 100 mV corresponding to abiotic oxidation or reduction of soil compounds. These abiotic inflexion points were also detected with similar intensity in the CVs performed after 7 and 20 days in both open‐circuit MERC (Fig. 2A) and closed‐circuit MERC (Fig. 2B). pol‐MERC (Fig. 2D) showed an inflexion point at 0.2 V (versus Ag/AgCl) after 7 and 20 days, which appeared as well in closed‐circuit MERCs but with considerably lower intensity. This inflexion did not appear in the open‐circuit MERC profile.

The increased signal of the current response (10‐fold between the open‐circuit MERC and closed‐circuit MERC) revealed the anode enrichment of electroactive microbial communities, which may be due to an increase in the cell density on the electrode surface or to an increase in the microbial electron transfer rate. The higher enrichment of electroactive microbial communities was confirmed by the measurement of anode potential in open‐circuit MERC at the end of the experiment (20 days), which reached −480 mV potential in pol‐MERC, comparing with open‐circuit MERC and closed‐circuit MERC systems where the potentials were −250 and −300 mV, respectively. Thus, CVs reveal a different redox interaction and microbial activity occurring on the electrodes under different treatments, which may explain the differences observed in terms of ATR biodegradation.

### ^14^C‐ATR mineralization under short‐term assay

**Figure 3:**
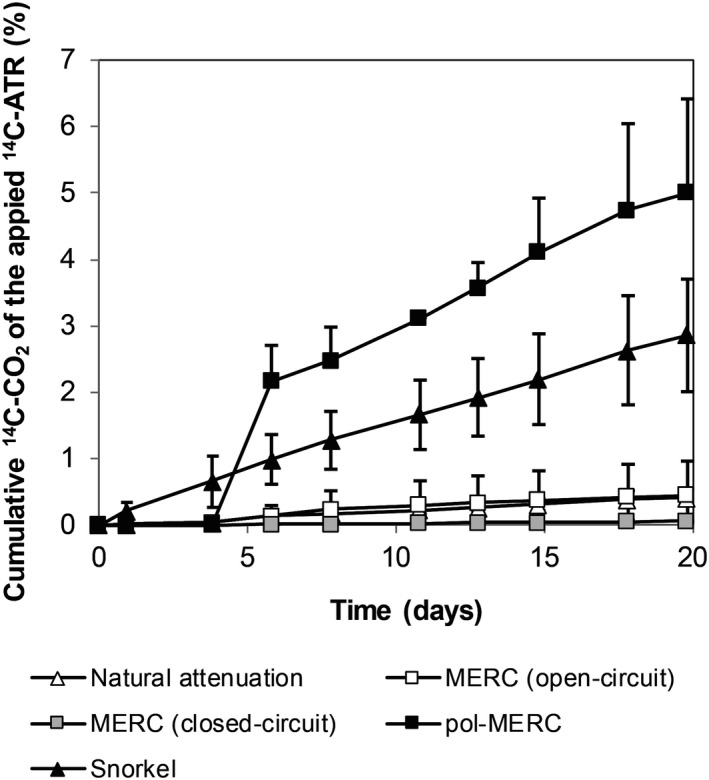
Cumulative mineralization of ^14^C‐ATR for short‐term assay (20 days) at different configurations (*n* = 3, SD). The error bars represent standard deviation.

Independent short‐term mineralization assays were performed to investigate the effect of the electrode's potential on the ^14^C‐ATR degradation. After 20 days of incubation, in the pol‐MERC cumulative mineralization reached 5%, the snorkel (Fig. 2C) configuration reached 3%, whereas under the rest of treatments mineralization was just 1% (Fig. 3).

The low cumulative mineralization under electrode‐free soil (Fig. 3) was an expected result given the absence of ATR application history in our soil (Schroll *et al*., 2006; Getenga *et al*., 2009; Ngigi *et al*., 2011). Additionally, the snorkel configuration revealed a higher mineralization response than MERC under either closed or open circuit (Fig. 3). Cruz Viggi *et al*. (2015) reported the stimulation of oxidative biodegradation of petroleum hydrocarbons in marine sediments using a snorkel configuration. Nevertheless, the highest mineralization occurred under the effect of a positive electrode potential (pol‐MERC). Thus, setting an electrode at +600 mV enhanced over 10‐fold the herbicide mineralization versus the electrode‐free soil.

### ^14^C‐ATR biodegradation pathway

Although ^14^C‐ATR was highly mineralized in our bioelectrochemical system, the analysis of the residual ATR metabolites suggests a possible biodegradation pathway. ATR biodegradation has been reported to follow chemical hydrolysis through *N*‐dealkylation and a dechlorination processes prior to ring cleavage (Fang *et al*., 2015). Either a single species or microbial consortia are responsible for reactions that involve three hydrolases genes *atzA*,* atzB* and *atzC* that encode for enzymes that dehalogenate and dealkylate atrazine in a stepwise fashion (Smith *et al*., 2005). Chemical hydrolysis results in the formation of hydroxyatrazine (HA‐ATR) and *N*‐dealkylation which results in the formation of deethylatrazine (DEA‐ATR) and/or deisopropylatrazine (DIA‐ATR) (Loos and Niessner, 1999). Complete degradation of ATR has been observed through continued hydroxylation of the triazine ring and the formation of ammeline, ammelide and cyanuric acid prior to ring cleavage, and finally to CO_2_ and NH_3_ (Wackett *et al*., 2002). It is known that ATR metabolites are relatively persistent in groundwater and soil/sediment (Enoch *et al*., 2007).

Analysis of soil samples revealed the presence of atrazine (ATR), deisopropylatrazine (DIA‐ATR), deethylatrazine (DEA‐ATR) and hydroxyatrazine (HA‐ATR) under all treatments (Table 1). After 7 days of pol‐MERC treatment, the ATR concentration was 2.6‐fold lower (0.70 μg g^−1^ of dry soil) than under natural attenuation (electrode‐free) (1.82 μg g^−1^ of dry soil). A similar situation occurred when pol‐MERC was compared with the rest of configurations. The differences in soil ATR levels between pol‐MERC and natural attenuation were maintained at ca. threefold after 20 days of incubation.

**Table 1:** Profile composition of the methanol‐extractable residues (metabolite concentrations appear as μg g^−1^ of dry soil) for different configurations and incubation times (7 and 20 days)

Atrazine and metabolites	Incubation time (d)	Electrode‐free soil (μg g^−1^ of dry soil)	MERC (open‐circuit) (μg g^−1^ of dry soil)	MERC (closed‐circuit) (μg g^−1^ of dry soil)	pol‐MERC (μg g^−1^ of dry soil)	Snorkel (μg g^−1^ of dry soil)
ATR^a^	7	1.829 ± 0.005	1.631 ± 0.010	1.595 ± 0.004	0.694 ± 0.018	1.279 ± 0.009
	20	1.026 ± 0.004	0.759 ± 0.008	1.026 ± 0.012	0.327 ± 0.023	0.845 ± 0.017
HA‐ATR^b^	7	0.103 ± 0.016	0.093 ± 0.007	0.092 ± 0.008	0.159 ± 0.009	0.081 ± 0.015
	20	0.147 ± 0.018	0.134 ± 0.008	0.123 ± 0.007	0.227 ± 0.014	0.131 ± 0.024
DEA‐ATR^c^	7	0.017 ± 0.006	0.022 ± 0.008	0.024 ± 0.004	0.026 ± 0.008	0.015 ± 0.005
	20	0.019 ± 0.005	0.020 ± 0.007	0.039 ± 0.008	0.045 ± 0.007	0.029 ± 0.005
DIA‐ATR^d^	7	0.006 ± 0.001	0.011 ± 0.004	0.014 ± 0.004	0.015 ± 0.006	0.005 ± 0.001
	20	0.008 ± 0.007	0.017 ± 0.006	0.019 ± 0.005	0.195 ± 0.013	0.016 ± 0.002

**a.** 1‐ 6‐chloro‐4‐*N*‐ethyl‐2‐*N*‐propan‐2‐yl‐1,3,5‐triazine‐2,4‐diamine.

**b.** 2‐(ethylamino)‐6‐(propan‐2‐ylamino)‐1H‐1,3,5‐triazin‐4‐one.

**c.** 6‐chloro‐2‐*N*‐propan‐2‐yl‐1,3,5‐triazine‐2,4‐diamine.

**d.** 6‐chloro‐2‐*N*‐ethyl‐1,3,5‐triazine‐2,4‐diamine.

Regarding ATR metabolites, HA‐ATR coming from the hydrolysis reaction was most abundant regardless of the treatment assayed. The highest concentration of HA‐ATR was detected in pol‐MERC‐treated soil, after 20 days of incubation. Interestingly, HA‐ATR formation was associated with anaerobic or oxygen‐limited conditions, like those found in flooded soil (Armstrong *et al*., 1967). Chung *et al*. (1996) reported a linear isotherm study where HA‐ATR showed sixfold higher adsorption to soil than ATR did (Chung *et al*., 1996). Regarding the DIA‐ATR metabolite, it was detected at high concentration in pol‐MERC‐treated soil, 10‐fold higher than the rest electrode‐assisted systems (open‐circuit MERC, closed‐circuit MERC and snorkel) and 24‐fold higher than the natural attenuation.

Although the snorkel treatment showed better mineralization response than MERC under either closed or open circuit, very similar metabolite patterns have been detected under each of these treatments. On the contrary, pol‐MERC has shown higher differences, mainly in the parent compound, HA‐ATRA and DIA‐ATRA, as has been already specified.

### Ecotoxicity assays reveal an effective clean‐up

**Figure 4:**
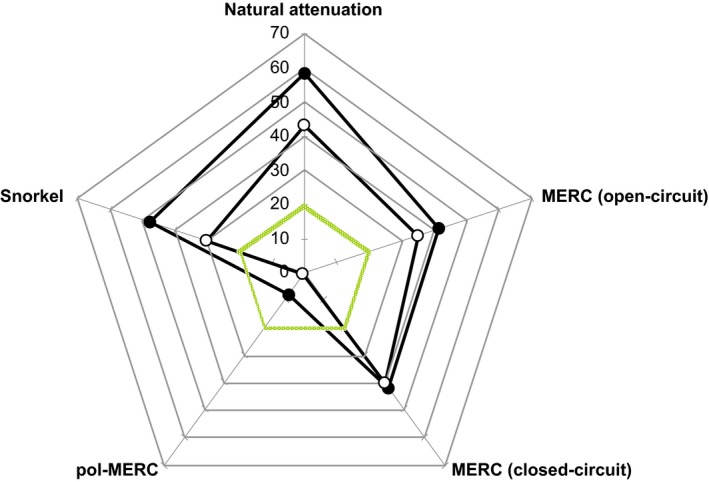
Toxicity of soils treated under different configurations. The toxicity values were represented as inhibition of *P. subcapitata* algal growth (%) at different incubation times: 7 days (●) and 20 days (○). Reference non‐toxic value was also provided (green line) to lower toxicity. The incubated soils under pol‐MERC treatment during 7 and 20 days showed an inhibition below the non‐toxic value, in contrast with the rest of the treatments, above non‐toxic level.

In order to evaluate the degree of soil restoration, ecotoxicological tests were performed after the incubation period (Fig. 4). Interestingly, after treating the soil using pol‐MERC for 20 days, soil extracts did not exhibit any inhibition of the algal growth in contrast with the inhibition (43%) shown under natural attenuation. Previous studies reported that ATR can exhibit a higher toxicity on algal species than its chlorinated primary metabolites do (Enoch *et al*., 2007; Ralston‐Hooper *et al*., 2009). This fact is consistent with the lower toxicity detected under pol‐MERC conditions, a treatment that revealed a high level of ATR mineralization (Fig. 4). On the contrary, after treating the soil using snorkel‐like devices, soil extracts still exhibited 30% of algal growth inhibition. In spite of the high mineralization shown under the snorkel treatment, the toxicity values after 20 days were similar to those obtained using MERC under either closed‐ or open‐circuit conditions (ca. 35–40%). So, only pol‐MERC exhibits global evidence to be designated as a favourable scenario for ATRA‐polluted soil remediation.

### Characterization of the ^14^C‐ATR mass balance

**Figure 5:**
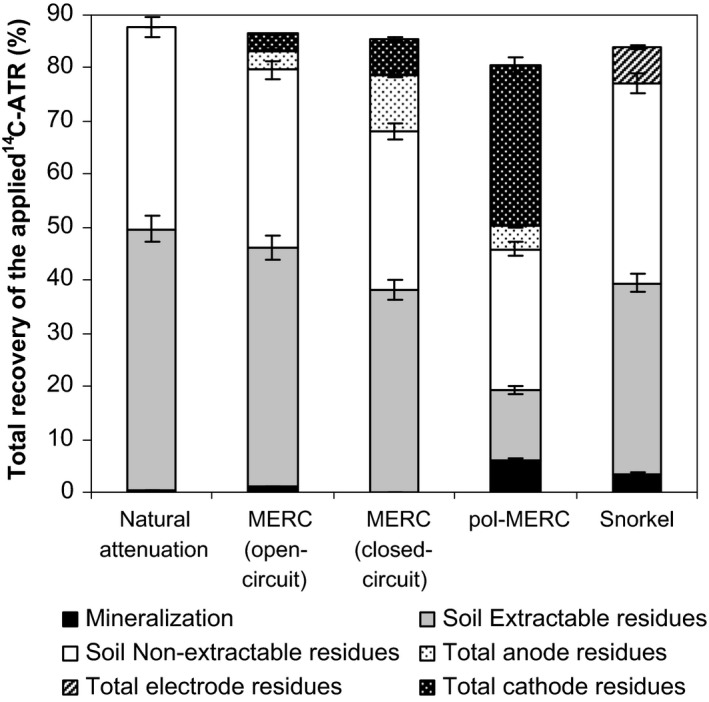
Mass balance of ^14^C‐ATR under the different treatments. The error bars represent the standard deviation for triplicate assays.

In order to give insight into the factors governing ATR mineralization, we performed a ^14^C‐mass balance with the different experimental set‐ups after 20 days (Fig. 5). We analysed the methanol‐extractable pesticide ^14^C residues (ER) from soil and electrodes together with the non‐extractable residues (NER). The ^14^C‐balances ranged between 82% and 88% of the applied ^14^C‐ATR as volatilization of dealkylated amino metabolites may have led to ^14^C losses (Loos and Niessner, 1999).

Extractable ^14^C residues (ER) levels showed a high variation regardless of the treatment after 20 days of incubation (Fig. 5). In pol‐MERC systems, where the cumulative ^14^C‐ATR mineralization was higher, the ER reached just 13%, while under natural attenuation the ER was slightly under 50%. Under closed‐circuit MERC and snorkel configurations, the ER reached intermediate values of approximately 37%. A similar distribution was observed for natural attenuation (38%), open‐circuit MERC (34%) and snorkel (37.6%) configurations for the NER in soil. Lower values were obtained when electrons flowed between electrodes, reaching 30% in closed‐circuit MERCs and 27% in pol‐MERC configuration.

The decrease in soil‐NER (Fig. 5) may be due to different metabolic pathways able to generate metabolites that have a lower affinity for soil than for electrodes. In that sense, DEA, DIA and HA‐ATR are strongly adsorbed to soil (Loos and Niessner, 1999), increasing the NER and decreasing the availability of the parent compound to continue with the mineralization pathway.

The percentage of extractable ^14^C residues (ER) from the electrode was negligible (around 0.15%), and thus almost the total fraction of electrode residues corresponded to NER. The percentage of NER from the electrodes was affected by the electron flow between electrodes. Thus, under open‐circuit conditions 7% of the applied ATR was attached irreversibly to the electrodes (3.5% for both anode and cathode, respectively). These values are similar to those obtained from ^14^C‐ATR adsorption assays (2.2%) on carbon felt. Total electrode residues increased considerably under treatments where electrons are flowing between electrodes (pol‐MERC), suggesting that ATR and its metabolites were attached to the electrodes as NER. Baskaran and Bolan (1998) (Baskaran and Bolan, 1998) determined that the atrazine molecule has a positive charge, and this could cause a major adsorption to negatively charged electrodes (Fig. 5) like the anode in the case of closed‐circuit MERC system (−250 mV) and the cathode in the case of pol‐MERC system (−150 mV).

### ^14^C‐ATR long‐term mineralization

**Figure 6:**
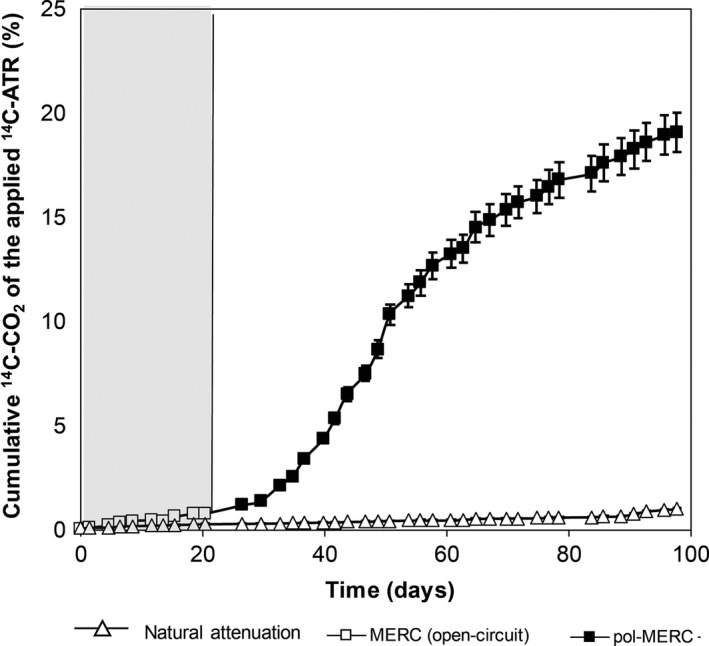
Monitoring of the cumulative mineralization of ^14^C‐ATR under different configurations for long‐term assay (100 days). At the first phase of the experiment (grey zone), MERC systems were operating under open‐circuit configuration (open squares) for 20 days. At the second phase of the experiment, MERC (open‐circuit) configuration was converted to a pol‐MERC (closed squares) operated at a poised anode potential of + 600 mV versus Ag/AgCl reference electrode. The cumulative herbicide mineralization under pol‐MERC increased to 20% of the initial ^14^C‐ATR, whereas the natural attenuation (soil without electrodes) (open triangles) was showing a negligible ATR mineralization. The error bars represent standard deviation.

Bioelectroventing atrazine‐polluted soil proved to toxicologically clean it after 20 days of treatment using a pol‐MERC. However, in order to evaluate the effect of bioelectroventing the polluted soil for longer periods, we prolonged the assay for 80 additional days. Our strategy was to convert two experimental open‐circuit MERC replicates just after finishing the short‐term assay, into pol‐MERC by shifting the electrode potential from negative (−250 mv versus Ag/AgCl) to positive (+600 mV versus Ag/AgCl). At the end of the long‐term assay, the cumulative herbicide mineralization under pol‐MERC increased to 20% of the initial ^14^C‐ATR whereas the natural attenuation was just 1% (Fig. 6). Therefore, the bioelectroventing enhanced the ATR mineralization capability of the soil native microbial community.

The neutral pH of our soil (6.8) and the flooding soil conditions of our assays allow CO_2_ to dissolve in water and establish an equilibrium with carbonic acid leading to negatively charged species as ${\text{HCO}}_{3}^{-}$ and ${\text{CO}}_{3}^{-}$ that could be adsorbed on the surface of positive polarized electrodes. In order to evaluate this hypothesis, after both short‐ and long‐term assays, the potential was shifted from positive potential (+600 mV versus Ag/AgCl) to negative (−300 mV versus Ag/AgCl) in pol‐MERC systems. The mineralization rate in pol‐MERC increased from 0.2% to 0.7% of the initial ^14^C‐ATR applied, indicating low adsorption of ^14^CO_2_ in the polarized anode.

Most of the research regarding biodegradation of ATR has been almost fully devoted to aerobic metabolism. Furthermore, ATR mineralization in soil depends on the pollutant exposure time. Actually, a significant ATR mineralization was reported by Getenga *et al*. (2009) by treating soils historically contaminated with the herbicide (Getenga *et al*., 2009). In contrast, a low mineralization (3–5% of the initial ATR) was reached using soils not previously exposed to ATR (Schroll *et al*., 2006; Ngigi *et al*., 2011). Similarly, low mineralization of ATR has been reported under anaerobic and strong reductive conditions (Nair and Schnoor, 1992; DeLaune *et al*., 1997; Seybold *et al*., 2001). The low mineralization of atrazine by native soil microbes has been attributed to the halogen on the pesticide ring, which impedes further microbial metabolism (Wackett *et al*., 2002). This is consistent with Crawford *et al*. (1998) studies in anoxic sediment slurries where a non‐significant mineralization was reported under denitrifying conditions (Crawford *et al*., 1998). Chung *et al*. (1996) reported 20% ATR transformation in anaerobic sediments after an incubation period of 30 weeks although mineralization was not reported (Chung *et al*., 1996). In spite of this oxygen‐associated limitation, our pol‐MERC was able to remove 93% of ATR in the first 20 days. In contrast, a rapid mineralization under denitrifying conditions was reported by *Pseudomonas* sp. strain ADP in aquifer sediments when it is amended with citrate and atrazine was presented in high concentration (Zophel *et al*., 1991; Shapir *et al*., 1998).

We concluded that using electrodes at a positive potential, we can overcome electron acceptor limitation and maximize ATR mineralization by ca. 20‐fold. Even more remarkable and according to our ecotoxicological assays, this so‐called *bioelectroventing* strategy was also successful for achieving an effective clean‐up of the soil able to restore the pre‐pollution conditions. Additionally, microbial electrochemical systems can be readily monitored and regulated by tuning electrochemical parameters, endowing it a good controllability, i.e. the produced electrical current can serve as a real‐time bioremediation indicator.

## Experimental procedures

### Chemicals

Uniformly ^14^C ring‐labelled atrazine (2‐chloro‐4‐ethylamino‐6‐isopropyl amino‐1,3,5‐triazine) (^14^C‐ATR) with a specific radioactivity of 1.2 kBq μg^−1^ and a radiochemical purity > 98.5% was purchased from GE Healthcare (Little Chalfont, UK). ^14^C‐labelled was mixed with non‐labelled atrazine to reach a final concentration of 2.5 μg μl^−1^ and a specific radioactivity of 63 Bq μg^−1^. This ATR mixed standard was applied to the soil. Non‐labelled atrazine and the metabolite standards of 2‐hydroxyatrazine (HA‐ATR), deethylatrazine (DEA) and deisopropylatrazine (DIA) were obtained from Sigma‐Aldrich (Fluka, Buchs, Switzerland). Scintillation cocktails (Ultima Gold XR and Ultima Flo AF, PerkinElmer, Waltham, USA) were obtained from Packard (Dreieich, Germany). All other chemicals and solvents were of analytical grade and purchased from Merck (Darmstadt, Germany).

### Soil

The soil material (aric anthrosol) was from an agricultural field (Hohenwart; latitude 48.250°, longitude: 11.567°, altitude 472 m) in Germany without ATR history and with an organic matter content of 0.99%. A complete physical–chemical analysis of this soil was previously reported by Grundmann *et al*. (2011) and the soil conductivity, 0.247 mS cm^−1^, was reported by Rodrigo Quejigo *et al*. (2016). Soil samples were taken at 0–20 cm depth and were stored in hermetically sealed plastic bags at −20°C for laboratory analysis according to guidelines of the Organization for Economic Cooperation and Development (OECD) from 1995. The soil samples were unfrozen following the incubation protocol reported by Folberth *et al*. (2009).

### Degradation experiments

#### ^14^C‐ATR application

About 0.1 ml of ATR mixed standard with a total radioactivity of 15.75 kBq was applied dropwise with a Hamilton syringe to an oven‐dry and pulverized soil sample of 3.5 g and mixed homogeneously for 1 min. After evaporation of the organic solvent (methanol), this 3.5 g of soil aliquot was mixed for another 2 min with 46.5 g of soil resulting in a total sample amount of 50 g of dry soil per experiment with a herbicide concentration of 5 (± 0.1) μg g^−1^ of soil (dry weight). The total spiked soil sample was transferred to the opaque incubation glass flask of the laboratory system described below, compacted to a soil density of 1.3 g cm^−3^ (its natural density in the fields) and adjusted to flooded conditions (water‐holding capacity + 50 ml of extra deionized water). Water evaporation was compensated weekly by the addition of deionized water.

#### Experimental set‐up and operational conditions for mineralizing ^14^C‐ATR

The mineralization experiment was conducted in a laboratory system built in approximation to the OECD guideline for testing of chemicals 304A (OECD, 1981). It consisted of opaque glass flasks (volume of 250 ml; neoLab, Heidelberg, Germany) that were closed with a rubber stopper (neoLab); at the bottom of the stopper, a plastic beaker with volume of 25 ml (VWR International, Darmstadt, Germany) was attached. The plastic beaker was filled with 10 ml of 0.1 N NaOH (Merck) to trap the ^14^CO_2_ resulting from the mineralization process. The trapping solution (NaOH) was collected three times per week, and the traps were filled with fresh NaOH solution; 2 ml of aliquots from collected NaOH was mixed with 3 ml of scintillation cocktail Ultima Flo AF (PerkinElmer, Rodgau, Germany) and the radioactivity was measured in a liquid scintillation counter (Tricarb 1900 TR; Packard). A hollow needle (neoLab) through the rubber stopper allowed the exposure of the cathode electrode to atmospheric oxygen without disturbing the anoxic environment surrounding the soil‐buried anode. Actually it is a common practice in microbial electrochemical systems to expose the cathode electrode to the presence of atmospheric oxygen given that oxygen reduction reaction is the dominant cathodic process. To prevent saturation of the NaOH solution with atmospheric CO_2_, a plastic reservoir (neoLab) filled with soda lime (Merck) was connected to the needle.

In order to evaluate the capability of soil microbial community to degrade ^14^C‐ATR under flooded conditions, the following four different conditions were set up (Fig. 2):

1. MERC (open‐circuit): Anode and cathode were disconnected. In this set‐up, electron transfer between electrodes could not flow, but electrodes still could supply to microbes a redox‐active surface to interact with. The redox potential of the anode was determined by the redox potential differences across sediment/water.
2. MERC (closed‐circuit): A MERC configuration where anode and cathode were connected by a cooper wire using 5Ω external resistor (R). This low resistor allows the system to operate at maximum current intensity, where the electron transfer between electrodes is maximum.
3. Snorkel (short circuit): Carbon felt electrodes were placed vertically, so half of the electrode was buried in the soil and the other half was in the flooded water body. The electroconductive material represents the complete redox potential spectrum across sediment/water/air. This configuration allows system to operate at maximum current intensity but, unlike configuration B, the redox potential is variable along the material.
4. pol‐MERC: operated at a poised anode potential of + 600 mV versus Ag/AgCl reference electrode (RE‐5B; BASi, UK) (+199 mV versus SHE) using a potentiostat (NEV2; Nanoelectra*,* Madrid, Spain).

All systems were compared with a system under flooded conditions in the absence of any conductive material acting as a control (natural attenuation). Indeed, electrode‐free soils were set up in the laboratory under the same water content, temperature and ^14^C‐ATR concentration as the rest of the electrode‐assisted assays.

Circular graphite felt conductive material (Mersen, Spain, 4.5 cm diameter, 0.5 cm thickness; 0.7 m^2^ g^−1^ of surface area) was used as the anodes and cathodes. It showed no ATR adsorption (Fig. S1 in SI) and very adequate mechanical properties to conform the system. Copper wires used for connections were sealed with a conductive epoxy resin (Circuit Works, Santa Monica, CA, USA) and isolated with a non‐conductive epoxy resin (Araldit Ceys, Spain) to a graphite rod (Mersen, Courbevoie, France), acting as a connector and inserted in the carbon felt. The electrodes were located at the bottom of the soil (anode) and above in the deionized water (cathode). The graphite did not show, as the carbon felt, any ATR adsorption (Fig. S1 in SI).

#### Mineralization assays

The mineralization of ^14^C‐ATR to ^14^CO_2_ was studied in a closed and aerated laboratory system, as described above. The soil samples were incubated at 30 ± 0.1°C for 20 days (short‐term assay). The first sampling of the NaOH trap was performed 24 h after ^14^C‐ATR additions, and then, sampling was performed three times per week. The cumulative ^14^CO_2_ was expressed as a percentage of the total applied ^14^C‐ATR.

After 20 days of incubation, three of the five open‐circuit replicates were sacrificed for soil analysis. The other two replicates were shifted into pol‐MERC conditions. So, the anodes were poised at +600 mV versus Ag/AgCl reference electrode (RE‐5B; BASi) (+199 mV versus SHE) using a potentiostat (NEV2; Nanoelectra). In the same way, three of the five natural attenuation replicates were sacrificed for soil analysis. Under both configurations, pol‐MERC and natural attenuation, ^14^C‐ATR mineralization was monitored for another 80 days (long‐term assay).

Short‐term parallel assays were also performed under the same conditions (five different configurations) although using non‐radioactive ATR. Each different configuration was set up with six experimental replicates; three were collected and extracted after 7 days and the rest after 20 days. The soil extracts sampled after 7 and 20 days were analysed by HPLC to determine the metabolite pattern and to conduct ecotoxicological test.

#### Analysis of radioactive soil extracts

After 20 days of incubation, soil aliquots and carbon felt electrodes were separately extracted with methanol in an accelerated solvent extractor (ASE 200; Dionex, Idstein, Germany) at 90°C, with a pressure of 10 MPa. Aliquots of 0.1 ml of each extract were mixed with 5 ml of Ultima Gold XR and measured by liquid scintillation counting to determine the extractable residues (ER).

After ASE, soil material was homogenized intensively. Three aliquots (each 250 mg) of each soil sample were placed into combustion cups and mixed with 7–8 drops of saturated aqueous sugar solution to guarantee a complete oxidation of the ^14^C. Carbon felt electrodes from each assay were cut in pieces and placed into combustion cups. Combustion was conducted with an automatic sample oxidizer 306 (Packard). ^14^CO_2_ was trapped in CarboSorb E (Packard) and mixed with Permafluor E (Packard) prior to scintillation counting.

#### Electrochemical characterization

The anode potential in MERC systems was continuously poised at + 600 mV versus Ag/AgCl (RE‐5B; BASi) using a potentiostat (NEV1‐3; Nanoelectra), and the resulting current was recorded every 60 s. Both anode and cathode potentials and the difference of potential between electrodes in pol‐MERC were recorded continuously with a multimeter (7700; Keithley instruments, Solon, Ohio, USA).

Cyclic voltammetry (CV) was performed at the beginning of the experiment (24 h) and after 7 and 20 days. To characterize the electrochemical activity of the soil microorganisms, a scan rate of 1 mV s^−1^ from the open‐circuit potential was applied by a potentiostat (NEV3; Nanoelectra).

#### ATR residue analysis in non‐labelled assays

At the end of the incubation periods (7 and 20 days), all soil samples from non‐labelled assays were extracted with methanol in an accelerated solvent extractor (ASE 200; Dionex) at 90°C, with a pressure of 10 MPa. The extracts were concentrated with a rotary evaporator to a volume of 2–3 ml and adjusted to 250 ml of distilled water for cleaning up with Isolate Triazine columns (500 mg; Separtis, Grenzach‐Wyhlen, Germany). After extraction, the SPE columns were dried under a gentle nitrogen stream and eluted with 10 ml of methanol. The eluate was concentrated to a volume of 1 ml with a rotary evaporator and further concentrated to dryness under a gentle nitrogen stream. All samples were resuspended to 200 μl; 20 μl of these samples was injected into a HPLC‐DAD (Varian 9040) with C18 Phenomenex Kromasil column (150 × 4.5 mm, 5 μm) and UV detection at 220 nm. The mobile phase consisted of 0.003 M of KH_2_PO_4_, pH 3 (A) and acetonitrile (B) at a flow rate of 0.8 ml min^−1^. The gradient program was as follows: T 0 min 20% A, T 10 min 38% A, T 24 min 75% A, T 29 min 75% A, T 33 min 20% A, T 40 min 20% A. Parent compounds and metabolites (HA, DEA and DIA) were identified by the comparison of their retention times with reference substances.

#### Algal test

The algal growth inhibition test was conducted with the atrazine non‐labelled soil extracts according to OECD Test Guide 201 (OECD 2008), using the green microalgae *Pseudokirchneriella subcapitata* (previously *Selenastrum capricornutum*) as reported previously (Boltes *et al*., 2012; González‐Pleiter *et al*., 2013). Microorganisms and culture media were purchased from MicroBioTest Inc. (Gent, Belgium). Following the manufacturer's instructions, algal beads of *P. subcapitata* were reconstituted with the provided dissolving matrix and were grown in 25 ml of the culture until reaching the logarithmic growing phase (3 days). Then, the cells were used as the inoculum for the toxicity test. Culture was performed at 22 ± 2°C under continuous light and stirring at 150 rpm. Cell growth was monitored by optical density (OD) of culture and was measured at 670 nm to control growth process (Shimadzu UV‐VIS 1800, Duisburg, Germany). Tests were started using the prescribed amount of 5 × 10^4^ cells per ml and were performed within 96‐well clear disposable microplates. Three replicates of each extracted soil sample, including negative control (atrazine‐free soil) and blank (algae‐free), were included. The dilution factor for the polluted wells was 0.93 (190 μl of extracted soil sample + 10 μl of growth media + 5 μl of alga culture containing 9.10^4^ cells ml^−1^). Plates were incubated during 3 days under the same conditions of light and temperature as the inoculum cultures but in the absence of stirring. The *in vivo* fluorescence emission of chlorophyll (excitation 450 nm; emission 672 nm) was measured daily using a FLUOROSKAN FL; Thermo Fisher (Barcelona, Spain). Finally, the percentage of growth inhibition in test samples was assayed in reference to the negative control assay.

## Conflict of interest

None declared.

## Supporting information


**Fig. S1.** ^14^C‐ATR adsorption in different electrode conductive material: graphite paper (A), graphite plate (B), carbon felt (C) and graphite rod (D).
