# Supplementary material for: Bioelectroventing: an electrochemical‐assisted bioremediation strategy for cleaning‐up atrazine‐polluted soils
Source: Microb Biotechnol. 2017 Jun 23;11(1):50–62. doi: 10.1111/1751-7915.12687 (PMC5743802; doi:10.1111/1751-7915.12687)
Supplement: Supplementary file 1 — Fig. S1. 14C‐ATR adsorption in different electrode conductive material: graphite paper (A), graphite plate (B), carbon felt (C) and graphite rod (D). [file MBT2-11-50-s001.doc]

**Bioelectroventing: an electrochemical-assisted bioremediation strategy for cleaning-up atrazine polluted soils**

Ainara Domínguez-Garay **^a,+^**, Jose Rodrigo Quejigo **^a,+^**, Ulrike Dörfler **^b^**, Reiner Schroll **^b^** and Abraham Esteve-Núñez **^ac*^**

**^a^** University of Alcalá. Alcalá de Henares, Madrid, Spain.

**^b^** Helmholtz Zentrum München. Múnich, Germany.

**^c^** IMDEA-WATER Parque Tecnológico de Alcalá. Madrid, Spain.

^+^ Ainara Domínguez-Garay and Jose Rodrigo Quejigo are equally contributors

**^14^C-ATR adsorption assays in different electro-conductive materials**

Several adsorption assays were conducted to investigate the adsorption of ^14^C-ATR on different conductive materials: graphite plate, graphite paper, carbon felt and graphite rod. 50 mL eppendorf tubes were filled with 39 mL of deionized water and ^14^C-ATR standard was added to vide a final concentration of 2.5 μg/μL and a specific radioactivity of 63 Bq·μg^-1^. Three replicates were conducted for each electrode material and the eppendorf tubes were shaken overhead continuously. At different time intervals aliquots of 1 mL were sampled to measure the radioactivity until the equilibrium for adsorption of ^14^C-ATR was reached. Each sample was mixed with 4 mL Ultima Gold XR and measured in a liquid scintillation counter (*Tricarb* 1900 TR, Packard, Dreieich, Germany). A control was performed under the same conditions but in the absence of material.

The higher adsorption was observed in graphite paper, that after 18 hours in incubation 86% of the applied ^14^C-ATR was retained in the material. The rest of materials showed lower adsorption behaviour than the graphite paper: 7.7% in graphite plate, 2.2% in carbon felt and 1.2% in graphite rod (Figure S1). Regarding to the results and considering the convenience physico-mechanical properties, the high surface area, and the inexpensive cost, the material used to conform the electrodes for mineralization assays was carbon felt and graphite rod.

**A**

**B**

**C**

**D**

**Figure S1***.* ^14^C-ATR adsorption in different electrode conductive material: graphite paper (A), graphite plate (B), carbon felt (C) and graphite rod (D)
